# Teachers’ judgment accuracy: A replication check by psychometric meta-analysis

**DOI:** 10.1371/journal.pone.0307594

**Published:** 2024-07-25

**Authors:** Esther Kaufmann

**Affiliations:** Research Methods, Assessment and iScience, Department of Psychology, University of Konstanz, Konstanz, Germany; The Open University of Israel, ISRAEL

## Abstract

Teachers’ judgment accuracy is a core competency in their daily business. Due to its importance, several meta-analyses have estimated how accurately teachers judge students’ academic achievements by measuring teachers’ *judgment accuracy* (i.e., the correlation between teachers’ judgments of students’ academic abilities and students’ scores on achievement tests). In our study, we considered previous meta-analyses and updated these databases and the analytic combination of data using a psychometric meta-analysis to explain variations in results across studies. Our results demonstrate the importance of considering aggregation and publication bias as well as correcting for the most important artifacts (e.g., sampling and measurement error), but also that most studies fail to report the data needed for conducting a meta-analysis according to current best practices. We find that previous reviews have underestimated teachers’ judgment accuracy and overestimated the variance in estimates of teachers’ judgment accuracy across studies because at least 10% of this variance may be associated with common artifacts. We conclude that ignoring artifacts, as in classical meta-analysis, may lead one to erroneously conclude that moderator variables, instead of artifacts, explain any variation. We describe how online data repositories could improve the scientific process and the potential for using psychometric meta-analysis to synthesize results and assess replicability.

## Introduction

The lack of replication studies in psychology—including in the subfield of educational research—has been highly criticized [1, 2]. In this study, we describe how psychometric meta-analysis can be used to assess replicability in the real world, in which it is not only impossible but also potentially undesirable to run an exact 1:1 copy of an existing study. Using the example of *teachers’ judgment accuracy* (i.e., the extent to which teachers’ assessments of their students correspond with assessments from another criterion, typically a standardized test score), we demonstrate how psychometric meta-analysis can be used to appropriately synthesize research findings across multiple studies and draw more accurate conclusions about the generalizability of results.

### Replication is not straightforward

If the result of a given study truly exists in the population, one should theoretically be able to observe it repeatedly, given the same conditions. However, demonstrating replicability is far from straightforward. For one thing, it is often impossible to perfectly replicate a study’s method and procedure (e.g., location, sample composition, materials). Therefore, it is difficult to interpret whether failure to obtain the same results in a 1:1 replication study means that an effect does not exist or whether the diverging results are due to variations in the procedure or method. Moreover, there is some controversy about whether re-running an exact copy of the original study is the best way to demonstrate replicability: if a result is “real,” one would thus also expect it to be relatively robust across, for instance, different assessment techniques (see [3–6]). Finally, studies based on less reliable measures and smaller samples are inherently more difficult to replicate than studies based on more reliable measures and larger samples, even if the hypothesis is correct [7].

### Using psychometric meta-analysis to assess replicability

Meta-analysis can be perceived as an evaluation of multiple replication studies on a given topic. Schmidt and Hunter [8] recognizing that “the perfect study is a myth” (p. 17) and introduced a *psychometric meta-analytical approach* ([8], see also [9], for the historical development of this approach). Similar to traditional “classical” meta-analysis, psychometric meta-analysis combines findings from a minimum of two studies focusing on the same subject. What sets Schmidt and Hunter’s psychometric meta-analysis apart is its ability to systematically adjust for variations in sample sizes among studies and measurement errors, along with other potential research artifacts (such as range restriction or dichotomization of continuous variables). It is important to note that sampling and measurement errors are inherent in all databases. By allowing researchers to systematically correct for different sources of *between-study* heterogeneity, psychometric meta-analysis enables researchers to assess whether a particular result may hold across multiple studies *without* running (imperfect) 1:1 comparison studies. Moreover, unlike 1:1 replication studies [3, 4, 10, 11], psychometric meta-analysis provides further insight into *why* the results of one study fail to replicate (e.g., due to low measure reliability, particular aspects of the task).

In this study, we show, through teachers’ judgment accuracy studies, the need for psychometric meta-analysis to assess replicability (see also [4]). There is an array of reviews on teachers’ judgment accuracy; hence, it is an ideal database for such an undertaking.

### Teachers’ judgment accuracy

According to Shavelson [12], “any teaching act is the result of a decision” (p. 144), highlighting the fundamental roles of judgment and decision-making in teaching. Teachers are constantly faced with a myriad of crucial decisions regarding students’ competency, motivation, and resource allocation. Essentially, judgment and decision-making constitute a significant aspect of a teacher’s daily responsibilities. Therefore, it is understandable that teacher education programs, such as those in Germany [13], also emphasize the importance of judgment and decision-making skills.

To effectively support their students’ learning and development, teachers must be adept at accurately assessing their competencies. Overestimating a student’s abilities can lead to an overly challenging learning environment, negatively impacting the student’s self-concept, motivation, and learning outcomes. Conversely, underestimating a student’s competencies can result in a learning environment that fails to sufficiently challenge them, leading to boredom and underperformance. Thus, inaccurate teacher judgments can hinder students from reaching their full potential and exacerbate inequalities in learning rates among students.

#### Reviews of teachers’ judgment accuracy

Given the critical necessity for teachers’ judgments to be accurate, a plethora of studies have delved into the accuracy of teachers’ judgments. The review conducted by Urhahne and Wijnia [14] sheds light on the diverse range of studies in this area, distinguishing between those focusing on relative and absolute judgment accuracy. To narrow down this expansive subject, we concentrate specifically on the relative judgment accuracy of teachers in the following sections. We posit that a systematic examination of these various forms of judgment accuracy is imperative, as they are interdependent (see [15]). Furthermore, within the realm of studies concerning teachers’ relative judgment accuracy, two distinct categories emerge: traditional teacher judgment studies and those rooted in social judgment theory [16–18].

*Meta-analysis of classical teacher judgment studies*. In classical studies on teacher judgment, the accuracy of teachers is described by the correlation (represented by *r*) between teachers’ assessments (e.g., a teacher’s evaluation of a student’s mathematical or verbal achievement) and an established criterion (e.g., a test score or student grades). We emphasize the significance of this approach due to several meta-analyses indicating that statistical predictions (e.g., a student’s test score) tend to surpass human judgment and decision-making in terms of accuracy across various domains. Empirical evidence supports the notion that tests serve as benchmarks for evaluating the accuracy of teachers’ judgments (see [19]; also [20]). Given the importance of this topic, numerous studies have been conducted, resulting in various meta-analyses (e.g., [15, 21, 22]). These meta-analyses have offered insights into the overall accuracy of teachers’ judgments. However, prior reviews have provided limited guidance on how to enhance accuracy. Moreover, most earlier reviews relied on classical meta-analytical methods, which are prone to methodological shortcomings (e.g., [5]).

To illustrate the application of psychometric meta-analysis in evaluating replicability, we conducted a psychometric meta-analysis focusing on the accuracy of teachers’ judgments.

Specifically, we concentrated on studies of teachers’ judgments of students’ academic *competencies*, as opposed to their more general cognitive abilities, which have been reviewed elsewhere (e.g., [23]). The ability to adapt learning environments to suit individual students’ needs and abilities relies heavily on teachers’ capacity to accurately assess their academic progress. Consequently, it is no wonder that researchers have shown an enduring interest in assessing teachers’ accuracy in evaluating students’ academic achievement and discerning the factors influencing the accuracy of such judgments.

To date, three meta-analyses have consolidated findings concerning the accuracy of teachers’ judgments regarding students’ academic achievements. The initial comprehensive examination by Hoge and Coladarci [21] synthesized data from 55 distinct judgment tasks across 16 studies. Their analysis revealed a median correlation of *r* = .66 (mean *r* = .65) between teachers’ judgments and students’ performance on achievement tests.

In the second review, Südkamp and colleagues [22] employed a classical quantitative meta-analytical approach to synthesize findings from 75 studies concerning the accuracy of teachers’ judgments, all of which were published post-1989 (thus excluding the studies covered in Hoge and Coladarci’s review, [21]). Compared to the earlier review by Hoge and Coladarci [21], Südkamp et al. [22] found a decreased correlation between teachers’ judgments and estimates of students’ academic achievement based on other criteria (median *r* = .53, mean *r* = .63).

A recent re-meta-analysis of Hoge and Coladarci’s [21] original review suggests that the accuracy of teachers’ judgments (initial meta-analysis: *r* = .65) has been underestimated, with the replicated meta-analysis showing higher correlations (*r* = .80 / *r* = .74 when accounting for a measurement error of .90). The study found no evidence that the accuracy of teachers’ judgments varied depending on whether tasks were classified as “classical” or aligned with social judgment theory (for detailed insights, refer to below), nor did they find evidence that teachers’ judgment accuracy depended on the subject (language, mathematics, or miscellaneous), whether a student had a learning disability, or grade level (see [15]).

*Meta-analysis of social judgment theory studies*. In the third review, Kaufmann et al. [24] followed the *social judgment theory* framework [16–18] to compare estimates of judgment accuracy in the educational domain (based on five tasks from four studies) with those from other domains (e.g., medical science, business; based on 45 tasks). According to *social judgment theory*, teachers’ judgment accuracy (*r*_*a*_) can be understood as a composite of various components summarized in the lens model equation [25–27].

More precisely, the lens model equation describes teachers’ judgment accuracy as contingent upon three key components: the consistency of teachers’ judgments (*R*_*s*_), the nature of knowledge teachers use in their evaluations (*G*: linear, additive, or, *C*: non-linear, multiplicative combination of information) is objectively “judgable” (*R*_*e*_). In their study, Kaufmann et al. ([24, 28]) identified an overarching correlation of *r* = .51 between (student) teachers’ judgments and other measures of student abilities (e.g., achievement tests).

*Summary and conclusions*. Importantly, research conducted by Kaufmann [15] illustrated that employing a psychometric meta-analytical approach resulted in higher estimates of judgment accuracy and lower estimates of between-study heterogeneity compared to the classical meta-analytical approach applied by Südkamp et al. [22]. Furthermore, Kaufmann [15] discovered no evidence indicating variations in teachers’ judgment accuracy based on whether tasks aligned with “classical” or “social judgment theory” frameworks. However, it is important to note that their investigation focused solely on a subset of teachers’ judgment accuracy data derived from a review conducted by Hoge and Coladarci [21], which encompassed studies predating 1989. Consequently, the question remains as to whether contemporary studies on teachers’ judgment accuracy may yield divergent findings [29].

### The need to evaluate the replicability of previous reviews of teachers’ judgment accuracy

#### Methodological aspects

*Aggregation bias*. There have been a number of developments in meta-analytic “best practices” since Hoge and Coladarci [21] and Südkamp et al. [22] were published, suggesting that there is a need to assess the replicability of previous reviews on teachers’ judgment accuracy [15, 29, 30]. One significant evolution pertains to the comparison between *aggregated person data* (APD) and *individual person data* (IPD). While the majority of meta-analyses rely on APD (e.g., the average judgment accuracy of a group of teachers and tasks within a specific study), Kaufmann et al. [5] explained in detail that APD may introduce an ecological fallacy. This stems from the fact that associations between two variables at the group (or ecological) level may differ from associations between similar variables measured at the individual level [31]. Despite the acknowledgment of ecological fallacies in meta-analysis (see also [32], p. 114), to the best of our knowledge, no empirical investigation has been conducted thus far concerning meta-analysis on teachers’ judgment accuracy. Consequently, we aim to address this gap in the following sections.

*Heterogeneity check*. As outlined in Kaufmann et al. [5], to identify distinctions in heterogeneity in APD meta-analysis, meta-regression is frequently applied to reveal any moderator variables. Moderator variables influence the relationship between two variables. For example, in the context of teachers’ judgment accuracy, students’ gender could serve as a moderator variable; for example, female students might be subject to less accurate judgments than their male counterparts or the other way around. Generally, employing summary data to represent individual participants poses challenges, as outlined in Kaufmann et al. [5], with additional insights provided by Lau, Ioannidis, and Schmid [33], Schmid, Stark, Berlin, Landais, and Lau [34], and Schmidt and Hunter [8] (p. 384).

While individual person data (IPD) meta-analysis presents a promising avenue to address this limitation of APD meta-analysis, to our knowledge, no empirical examination through IPD meta-analysis of classical teacher judgment accuracy values has been undertaken thus far.

*Publication bias*. Finally, although still somewhat controversial, numerous methodological experts now advocate for assessing *publication* bias, also recognized as a “file-drawer problem” (i.e., the tendency for studies with non-significant results or with low correlation values to go unpublished or remain in the file drawer, as described [8, 35]). It is important to note that publication bias is also associated with additional biases, such as language bias and availability bias, as underscored by Rethlefsen et al. [36], Rothstein [35], and Song et al. [37]. Until relatively recently, the detection of publication bias was primarily reliant on graphical methods; however, since then, more sophisticated methods have been introduced [38]. It is therefore surprising that none of existing reviews examining teachers’ judgment accuracy regarding students’ academic competencies considered whether publication bias might have skewed their results. The recent re-analysis conducted by Kaufmann [15] suggests the presence of publication bias within the database used by Hoge and Coladarci [21]. Because studies by the supplementary review of Hoge and Coladarci [21] by Südkamp et al. [22] do not consider any publication bias estimation, it is uncertain whether this result can be replicated with an updated and expanded database. Consequently, there is a need to replicate the estimation of publication bias using current studies within the field, rather than relying solely on one review considering studies up to 1989; therefore, this single review does not adequately represent the entire study sample.

*Comparison of APD and IPD meta-analysis*. Although IPD has traditionally been viewed as the gold standard of meta-analysis [39] to address the limitations of APD, we also underscore the drawbacks of the time and cost associated with conducting an IPD meta-analysis. For additional disadvantages of APD relative to IPD meta-analysis, we mention that there is no control of the used statistical data combination or carrying out detailed data checks (see e.g., [40]).

Kaufmann et al. [5] (see also [41]) argue that Internet-based research offers a potential solution to these challenges, given advancements in technology. We refer to Kaufmann et al. [5] for additional drawbacks of APD meta-analysis and how to overcome them.

Therefore, we anticipated that, with recent technological advancements facilitating the integration and storage of IPD from multiple studies, IPD meta-analysis would emerge as a promising contemporary approach that warrants consideration. Since all previous reviews on teachers’ judgment accuracy have relied on aggregated data, the potential influence of aggregation bias on their findings remains unknown. Additionally, with the increasing feasibility of technology-based research, there has been a rise in the reporting of individual data, which is essential for conducting an IPD meta-analysis.

In situations where APD must be used, researchers now have the option to employ Schmidt and Hunter’s psychometric approach [8] to adjust for differences between studies, particularly variations in sample size and measurement reliability, which can potentially introduce bias into the meta-analytical results [42]. Kaufmann’s [15] re-analysis of the studies included in Hoge and Coladarci’s [21] review, using a contemporary psychometric meta-analytic approach, underscored the necessity of employing modern techniques to re-evaluate the findings of additional meta-analyses in the field (such as [22]), thus enhancing the generalizability of meta-analytical estimated teachers’ judgment accuracy.

#### Topic-related aspects

Apart from methodological concerns, there are also a number of topic-related reasons that warrant an evaluation of the replicability of previous reviews. Initially, numerous classical (i.e., non-lens model) studies investigating teachers’ judgment accuracy have been published since the most recent review. Moreover, although each review revealed that estimates of judgment accuracy varied considerably across studies [21, 22, 24], their results regarding potential *moderators* were not extensively informative.

Hoge and Coladarci [21] and Südkamp et al. [22] both examined various judgment characteristics as potential moderator variables. Their potential moderator variables include whether judgments are informed or uninformed (i.e., if teachers are aware of the evaluation criterion), the specificity of judgments (such as ratings, rankings, and estimations regarding correct responses), whether judgments are norm-referenced or peer-independent (i.e., if they are compared within the students’ class), and the domain specificity of judgments (whether they pertain to overall academic achievement or specific achievements within subjects).

Südkamp and colleagues [22] found evidence that only *informed judgment* and *judgment specificity* significantly influenced teachers’ judgment accuracy (i.e., higher judgment accuracy for informed judgments relative to uninformed judgments and for very specific judgments relative to general judgments). Contrary to their expectations, Südkamp et al. [22] found no evidence of other potential moderator variables, including judgment subjects **(**language, arts, or mathematics), and they were unable to check whether students’ gender moderated teachers’ judgment accuracy due to insufficient data. Hoge and Coladarci [21], while not specifically examining the subject as a potential moderator variable, covered a variety of judgment tasks in their review, such as judgments of students’ reading comprehension and mathematical problem-solving achievement.

### Current study

Changes in meta-analytic best practices, the publication of new studies, and the use of new technology for gathering and reporting more individual data and unanswered questions about potential moderators together indicate that there is a need to assess the replicability of previous reviews of teachers’ judgment accuracy. Therefore, in the current study, we conducted a psychometric meta-analysis of teachers’ judgment accuracy to re-evaluate the results of previous reviews using more up-to-date methods. We also conducted a new review based on an up-to-date database. Thus, our study not only informs research on teachers’ judgment accuracy specifically but also serves as an example of how psychometric meta-analysis can be used to assess the replicability of reviews and studies in a particular field more generally.

In line with current meta-analytic best practices, we first aimed to compare the results of an IPD and APD meta-analysis of teachers’ judgment accuracy. We then used a modern meta-analytic approach to re-analyze the data included in the different existing reviews [21, 22, 24]; as well as synthesize the results of all available studies on teachers’ judgment accuracy published up until 2018.

We estimated publication bias, and we (re-)assessed whether teachers’ judgment accuracy depends on students’ gender and judgment subject (language, math). We also examined whether publication year was associated with estimates of teachers’ judgment accuracy, as recent increases in teacher development courses may have stimulated improvements in teachers’ judgment accuracy.

Our specific research questions were as follows:

1. How accurately do teachers judge students’ academic abilities?
2. Which *theoretically meaningful factors* explain variations in (i.e., moderate) teachers’ judgment accuracy (subject area, students’ gender)?
3. To what extent do *methodological factors* explain variations in estimates of teachers’ judgment accuracy (aggregation bias, study artifacts, publication bias)?

## Methods

### Literature search

We conducted a literature search in line with the procedure from Südkamp et al. [22]. For details, we refer to S1 File (see also [36]).

### Databases

The complete database consisted of 122 studies: *k*_*HC*_ = 16 studies included in the review by Hoge and Coladarci ([21], HC), *k*_*SUED*_ = 75 studies included in the review by Südkamp et al. ([22], SUED), *k*_*LENS*_ = 5 tasks (from 4 studies) included in the review by Kaufmann et al. ([24], Lens), and *k*_*NEW*_ = 26 newly identified studies on teachers’ judgment accuracy published between 2009 and 2018 (for a list of all considered studies, see S2 File). All data for our study are available at the Open Science Framework (OSF): https://osf.io/54y98/?view_only=5bafa62c7e644cafbbec94512c505d21.

### Coding

We coded each study according to publication and study characteristics. For details, we refer to S3 File.

### Missing information

Where necessary, we contacted the first study authors to obtain missing (i.e., unpublished) information. In March 2019, we sent an e-mail to the 30 first authors of the studies included in Südkamp et al. [22], for which e-mail addresses were available. Only 23% (*k* = 7) of the first authors contacted responded to our message. Six authors apologized for not having the requested information. We were able to obtain missing information from one author after signing a contract.

### Study characteristics

#### Publication year and origin

While earlier studies were mostly from the United States, more recent studies were mainly from Europe (for details, refer to S4 File).

#### Individual person data

No classical teachers’ judgment accuracy study explicitly considered IPD. IPD was available for just one study, the study by Hoge and Butcher [43] in Hoge and Coladarci [21] and five tasks (four studies) in lens model studies. In the lens model studies, IPD was available for 93 individuals who made between 25 and 120 judgments (for details, see [28]). Hence, there is a difference in reporting IPD between lens model studies and classical teachers’ judgment accuracy studies.

#### Student samples

The student sample size ranged between 12 [44] and 9,650 students [45] (*M* = 704 students). Information on the gender composition of the student sample was available for approximately half of the studies (*k* = 63, 51.6%). The reported proportion of female students ranged from 0% to 80% (*M* = 49.8%).

#### Teacher samples

Information about teacher sample size was available for *k* = 91 (75%) studies (15 in [21], 49 in [22] 2012, 3 in [24], one study was written in Hebrew. In this study, we had to assume that the teacher sample size was missing, and 24 new studies). The reported teacher sample size ranged from one [46] to 3,483 [47] (*M* = 102).

#### Subject area

Table 1 displays the number of studies for which information on teachers’ judgment accuracy was available regarding students’ academic abilities in language, math, or language and math. Most studies provided information on teachers’ judgment accuracy with regard to students’ language achievement (44%), students’ language *and* math abilities (35%), and students’ math achievement (12%). The distribution was similar across each database. Nine studies (7%) required teachers to judge students’ academic achievement in language and math but did not report subject-specific data and were hence not included in our subject-specific meta-analysis (see Table 4).

**Table 1:** Number of studies with subject-specific information on teachers’ judgment accuracy.

Database (*k*)	Subject Area			
	Math	Language	Both	Other
HC: [21] (16)	1	6	9	0
SUED: [22] (75)	8	32	35	0
NEW: New studies (26)	4	14	8	0
LENS: [24] (5)	1	2	0	2
Total (122)	14	54	52^1^	2

*Note*. HC = Hoge and Coladarci [21] database; SUED = Südkamp et al. [22] database; NEW = studies published between 2009 and 2018; LENS = lens model studies included in Kaufmann et al. [24]; *k* = number of studies within the database.^1^ Nine of the 52 studies required teachers to judge students’ academic achievement in language and math but did not report subject-specific data.

#### Reliability

Table 2 displays the available reliability information. More studies provided information regarding the reliability of the criterion (*k* = 71, 58.2%) than regarding the reliability of teachers’ judgments (*k* = 47, 38.5%). For more information, please see S5 File.

**Table 2:** Descriptive statistics of the available reliability information.

Reliability information	Judgments					Criteria				
	*N*	*M*	*SD*	*min*	*max*	*N*	*m*	*SD*	*min*	*max*
	47	0.89	0.09	0.67	0.99	71	0.86	0.08	0.64	0.99

*Note*. *N* = number of reliability values.

### Analytical strategy

We were unable to conduct an IPD meta-analysis due to insufficient data from classical teachers’ judgment studies. We used the R-program [48] and specifically the psychmeta [49, 50] and metafor [51] packages to conduct our Schmidt and Hunter [8] analyses.

#### Bare-bones meta-analyses

To check the results of the original reviews, we first ran “bare-bones” meta-analyses on the studies included in each dataset separately, on all classical (i.e., non-lens model) studies, and finally on the complete database. Because we used the Schmidt-Hunter approach [8], we assumed a random-effects model. Such a random-effects model considers differences among participants and studies. In line with Schmidt and Hunter [8], we weighted studies by the number of students to adjust for sampling errors. Bare-bones meta-analyses adjust only for differences in sample size across studies [22].

In our analysis, the number of studies or tasks is indicated by “*k*”, and the judged students are indicated by “*N*”. In our bare-bones meta-analyses, the sample size weighted observed teacher judgment accuracy correlation is represented by *r*_*ob*_, with SD_ob_ showing the observed standard deviation.

We re-ran the results of the bare-bones meta-analysis of the complete sample and checked our results with different sensitivity analyses, for example, excluding outliers [52]. We used a forest plot to graphically compare the results of the studies in the complete database. We used forest plots and influential case diagnostics to identify outlier studies. We used the trim-and-fill method [38] and Kendall’s tau to estimate a possible publication bias [53]. Significant Kendall’s tau values indicate evidence of publication bias.

#### Moderator analyses

To examine potential moderators, we conducted a bare-bones meta-analysis on the subsample of studies for which information on judgment accuracy was available for both language and math and subject-specific information on the gender composition of the student sample (*k*_*Subject/Gender*_ = 31). To examine the potential interactions between subject area and student gender, we compared the results of the bare-bones meta-analysis of the subsample of studies with information on teachers’ judgment accuracy regarding students’ achievement in language and math and the proportion of female students in the study. We divide the dataset into three groups representing studies with less than 45% of female students in their studies or more than 55% of female students in their studies, and one group with the portion of female students between 45% and 55%. Again, we checked our analyses with several sensitivity analyses (e.g., outlier analyses).

For our moderator analyses, we used the 95% confidence interval (CI) computed around teachers’ judgment accuracy (*r*_*ob*_) for a heterogeneity measure. If the confidence intervals for our analyses, for example, on teachers’ judgment accuracy on students’ math and language competence, did not overlap, then the suggested moderator variable (subject field) seemed to have an impact. In addition, we computed the 80% credibility interval (CV). The difference between confidence and credibility interval is important (for further information, see [54]); contrary to the confidence interval, the credibility interval does not depend on sampling errors (or other artifacts) because variance due to artifacts has been removed from the estimation. Hence, credibility intervals provide useful information for the practice, as they focus on the corrected variability of the results and show whether this interval includes a positive aggregated value. In line with Morris’s recommendation [55] (p. 246), we report both intervals in our meta-analyses because it also gives us additional generalizability information:

The confidence interval provides the expected range of results if the mean effect size were to be calculated on a new sample of studies with the same characteristics (e.g., the same number of studies and sample sizes) as the current meta-analysis. The credibility interval, on the other hand, provides the expected range of results for an individual study randomly sampled from the population of potential studies, where the effect size is estimated without sampling error. Confidence and credibility intervals provide distinct and useful information, and both should be reported in a meta-analysis.

#### Psychometric meta-analyses

In the third step, we ran a psychometric meta-analysis because many artifacts can cause wide variations in the observed effect sizes. As we introduced before, in psychometric meta-analyses there are a palette of artifact corrections that sometimes occur in every study, like sampling measurement errors or dichotomization (the latter occur not in every study). We corrected for artifacts that always occurred in each study, namely for sampling and measurement errors. Sampling and measurement errors are relevant in each study and may have an impact on the overall results. We highlight that we also checked the study sample for dichotomization of a continuum variable and only found one study [56] in which dichotomization was reported. According to Schmidt and Hunter [8] (p. 43), the correlation in this study was underestimated at least 20%. Because only one study was revealed in our study sample, we argue that the overall results were not impacted by this study and used the original correlation value. Moreover, it seems that dichotomization is not relevant in studies on teachers’ judgment accuracy. We argue that there was no evidence that artifacts such as range restriction were relevant in this study sample. In our meta-analyses we used all artifact corrections that seemed to be relevant, but also for which data were available. We highlight that the construct validity of teachers’ judgment or the achievement test was not the scope of our psychometric meta-analyses due to missing data for such a correction.

In sum, we used information about the reliability of teachers’ judgments as well as the reliability of the criterion (e.g., achievement test) to correct for measurement error. As displayed in Table 2, not all studies reported reliability values. We therefore used an artifact distribution compatible with the Schmidt and Hunter approach [8] to correct for study artifacts. This approach entails using the available reliability data to estimate missing reliability data.

We then ran our psychometric meta-analysis and compared the impact of the corrections to the results. Therefore, we first show the results after correcting for sample bias (bare-bones meta-analyses), and then a complete psychometric meta-analysis was conducted. To check the impact of measurement error on teachers’ judgments, a psychometric meta-analysis was conducted in which teachers’ judgments were not corrected for measurement error, and one in which the evaluation criterion (achievement tests) was not corrected for measurement error. Thus, we argue that the differences between these three psychometric analyses will give us a sense of the impact of measurement error on the accuracy of teachers’ judgments.

In our psychometric meta-analyses, the value ρ is the estimate corrected for artifacts, and hence, teachers’ judgment accuracy corrected for artifacts; SDρ estimates the variability across studies or tasks while accounting for study-to-study (task-to-task) differences in the quality of teachers’ judgments. Again, we calculated the 95% confidence interval (CI) and the 80% credibility interval (CV). Please be aware that, compared to the 80% credibility interval reported in our bare-bones meta-analyses section, within the psychometric result section, the 80% credibility interval is not only corrected for sampling error, but also for measurement error.

## Results

### Outlier diagnostics

Based on the forest plot and influential case diagnostics, we identified three outlier studies (see S6 File).

### Publication bias

There was no indication of publication bias according to either the trim-and-fill method or Kendall’s tau in any of the analyzed datasets (HC: 0.05, *p* = .82; SUED: 0.01, *p* = .83; NEWS: -0.07, *p* = .63; LENS: 0.04, *p* = .48; complete database: 0.02, *p* = .72).

### Bare-bones meta-analyses

Table 3 displays the results of the bare-bones meta-analysis. At first glance, the estimates of teachers’ judgment accuracy based on the different datasets Hoge and Coladarci ([21] HC), Südkamp et al. ([22] SUED, contrary to the analysis by [22], we considered all studies and did not exclude the studies by [47, 57]), and lens model studies (LENS) were largely in line with the results originally reported by the respective reviews. The bare-bones meta-analysis of the complete dataset indicated that teachers’ judgments were moderately correlated (*r*_*ob*_ = .58, SD_obj_ = 0.12) with students’ achievement according to the criterion. The correction for sampling error by our bare-bones meta-analysis explained 4% of the variance in estimates of teachers’ judgment accuracy between studies. Excluding the three outlier studies did not change the results (*r*_*ob*_ = .58, SD_ob_ = 0.12, complete overlap of the 95% CI). Across reviews, there was no considerable heterogeneity in estimates of teachers’ judgment accuracy, and this heterogeneity was also low across the samples of classical and lens model studies (*r*_*ob*_ = .58, SD_ob_ = 0.12 and *r*_*ob*_ = .43, SD_ob_ = 0.15, overlap of the 95% CI), respectively. Due to the small sample size of lens model studies, however, results regarding the heterogeneity of the sample and the averaged teacher judgment accuracy value of lens model studies should be interpreted with caution. The 80% CV also indicates heterogeneity within each dataset.

**Table 3:** Results of the bare-bones meta-analyses of the different databases.

Database	*K*	*N*	*r*_*ob*_	*SD*_*ob*_	95% CI	80% CV
HC: [21]	16	6141	.66	0.07	.62–.69	.57–.75
Sued: [22]	75	38873	.56	0.10	.54–.58	.43–.69
New: New studies	26	40481	.59	0.14	.54–.65	.40–.78
Classical studies	117	85495	.58	0.12	.56–.60	.42–.74
Lens model studies	5	383	.43	0.15	.24–.62	.24–.61
Overall	122	85878	.58	0.12	.56.–.60	.42.–.74
Overall excluding outliers	119	85080	.58	0.12	.56.–61	.43.–.74

*Note*. *k* = number of studies; *N* = number of judged students; *r*_*ob*_ = observed mean effect size (teachers’ judgment accuracy); *SD*_*ob*_ = *SD* of observed effect size; 95% CI = 95% confidence interval; 80% CV = 80% credibility interval; HC = Hoge and Coladarci [21] database; SUED = Südkamp et al. [22] database; NEW = updated database, studies published between 2009 and 2018; Classical studies = non-lens model studies, studies included in Hoge and Coladarci [21], Südkamp et al. [22], and the updated dataset; LENS: all lens model studies.

### Moderator analyses

Table 4 displays the results of the bare-bones meta-analyses of the subject-specific teachers’ judgment accuracy and that were compared with the same dataset across subject fields for which subject-specific data were available (*N* = 31 studies). Our analyses across subject fields confirmed our previous analyses showing that this subset represents the overall dataset well. Within this data subset of 31 studies, teachers judged students’ mathematical achievement (*r*_*ob*_ = .59, range: 95% CI: .53–.65) and students’ language achievement (*r*_*ob*_ = .6, 95% CI: .56–.63), with no significant difference. We re-ran the analyses with several sensitivity analyses (e.g., outlier and publication bias estimations), which all confirmed our results.

**Table 4:** Subject-specific estimates of teachers’ judgment accuracy based on the results of the bare-bones meta-analyses of the subsamples of studies with subject-specific information on the gender composition of the student sample.

Moderators	*k*	*N*	r_ob_	SD_ob_	95% CI	80% CV
Language	31	19488	.60	.10	.56–.63	.47–.72
Below 45% girls	5	829	.64	.13	.48–.79	.46–.82
Between 45–55% girls	21	16422	.59	.11	.54–.64	.45–.72
Above 55% girls	5	2237	.63	.06	.56–.70	.56–.71
Math	31	19488	.59	.15	.53–.65	.39–.79
Below 45% girls	5	829	.56	.17	.35–.77	.32–.80
Between 45–55% girls	21	16422	.60	.16	.52–.67	.39–.81
Above 55% girls	5	2237	.56	.12	.41–.70	.38–.73
Across subject fields	62	38976	.59	.13	.56–.63	.43–.76
Below 45% girls	10	1658	.60	.15	.49–.70	.41–.79
Between 45–55% girls	42	32844	.59	.13	.55–.63	.42–.76
Above 55% girls	10	4474	.59	.10	.53–.66	.47–.72

*Note*. *k* = number of studies; *N* = number of judged students; *r*_*ob*_ = observed mean effect size (teachers’ judgment accuracy); *SD*_ob_ = *SD* of observed effect size; 95% CI = 95% confidence interval; 80% CV = 80% credibility interval.

We also checked whether the proportion of females in the dataset influenced teachers’ judgment accuracy. Our analysis did not confirm any differences due to the number of females across subject fields (see Table 4). Our check within subject fields confirmed that there seemed to be no differences in teachers’ judgment accuracy within subject fields, because teachers’ judgment accuracy ranged from .64 to .56 with overlapping confidence intervals. Therefore, our results are tentative, implying that teachers do not judge boys and girls differently within different subject fields. Our 80% CV was still large, meaning that heterogeneity was not explained by our presented analyses.

### Psychometric meta-analyses

Table 5 displays the results of the psychometric meta-analyses when assuming the correction of only one variable or both (see Table 2).

**Table 5:** Results of the psychometric meta-analysis corrected for reliability (*k* = 122, *N* = 85878).

Corrections	Psychometric Meta-Analysis			
	ρ	*SD*_*ob*_	95% CI	80% CV
Both variable^1^	.65	0.14	.62–.67	.48–.81
Achievement test	.62	0.13	.59–.64	.45–.78
Teachers’ judgments	.60	0.13	.58–.63	.44–.77

*Note*. ρ = mean corrected (teachers’ judgment accuracy); *SD*_*ob*_ = *SD* of observed effect size; 95% CI = 95% confidence interval; 80% CV = 80% credibility interval^1^; both variables are corrected for study artifacts, teachers’ judgments, and the achievement test.^2^ Only one variable was corrected for study artifacts.

Correcting for the reliability of teachers’ judgments and the criteria based on the available reliability values and hence several artifacts increased the estimate of teachers’ judgment accuracy (ρ = .65; see Table 5). By the psychometric meta-analyses, there are now 10.5% of the variance in estimated teachers’ judgment accuracy between studies explained, 4% due to sampling error and 6.5% due to other artifacts. If we compare the 95% CI of our bare-bones meta-analyses with the complete psychometric meta-analyses, then the two 95% CIs do not overlap. Hence, we also argue that this result shows the impact and need for a psychometric meta-analysis, although our 80% CV reveals that there is still some heterogeneity within our dataset that needs further exploration.

For a sensitivity check of our results through a complete psychometric meta-analysis, we also present the impact of measurement errors. In the first run, we only corrected the measure of teachers’ judgment. We assumed no measurement errors in any of the achievement tests used to evaluate the teachers’ judgments (criteria). In this case, teachers’ judgment accuracy decreases (ρ = .60), and the explained variance in the estimated teachers’ judgment accuracy between studies was reduced to 6.7%. However, because we assumed no measurement error in any achievement test used in our analyses, which is unrealistic, we see these analyses also as a lower bound of teachers’ judgment accuracy. To complete our analyses in the following, we assumed that we measured teachers’ judgments as 100% reliable but considered measurement error in our tests for the evaluation of teachers’ judgments. Compared to the complete psychometric meta-analysis, in this psychometric meta-analysis, teachers’ judgment accuracy also decreased (ρ = .62), but 7.8% of the variance in the estimated teachers’ judgment accuracy between studies is still explained.

## Discussion

Using the example of teachers’ judgment accuracy, we demonstrated how psychometric analysis can be used to increase the accuracy of assessing the replicability of previous studies and even previous meta-analyses. Because researchers can systematically adjust for sampling, measurement error, and other artifacts, the results of psychometric meta-analyses are less ambiguous than the results of (by default imperfect) 1:1 replication studies and demonstrate the extent to which results are robust across different measures and samples. Unlike 1:1 replication studies, psychometric meta-analyses also provide clues as to *why* the results of a particular study may not be replicable (e.g., due to less reliable measures). In our meta-analysis, we show that at least 10.5% of the explained variance is associated with artifacts, reducing the success of replication by 10.5% when there is no artifact correction considered.

Our analysis not only addresses the imperative to critically reassess existing meta-analyses [58] but also demonstrates the significance of systematically evaluating the body of studies pertaining to a specific subject. For instance, we observed that studies on classical teachers’ judgment accuracy failed to compile IPD or report reliability values compared to lens model studies. Initially, lens model researchers advocated an idiographic-statistical approach to analyze judgments. Hence, the available IPD by lens model studies may be explained by a different research tradition. The lack of data from classical studies on teachers’ judgment accuracy impeded our ability to conduct a meta-analysis according to current best practices considering classical and lens model studies.

We attempted to obtain missing information by contacting the relevant first authors via email. This process was not very successful (see also [59], revealing the same problems with medical data). Utilizing online data repositories presents a notably more efficient and robust approach to compiling data on a specific topic. Ideally, researchers would begin a research project by registering their study on an online platform similar to existing study pre-registration platforms (e.g., https://cos.io/prereg/). The platform would guide researchers on the necessary data to collect (such as IPD, reliability data, sample sizes, and specific data that could address gaps in the literature). Upon completing their studies, the researchers uploaded their data to a data repository. The platform could be designed to automatically conduct meta-analyses of the contributed data, constituting what is termed a “living systematic review” [60]. Such platforms would not only radically streamline the research process but also potentially reduce publication bias by providing an outlet for researchers to report “non-results” that may encounter difficulty in traditional academic journals. Users of the platform, both within and outside academia, would have access to an updated synthesis of research and be informed about any existing gaps in the literature.

The outcomes of our analysis offer significant insights into the realm of teachers’ judgment accuracy. First, our results suggest that failure to correct for several artifacts (e.g., sample size, measurement error) leads to skewed (under)estimates of teachers’ judgment accuracy. In our review, we not only address the most prevalent artifacts but also scrutinize the study samples, revealing that certain artifacts are not pertinent in current studies on teachers’ judgment accuracy, such as dichotomization and range restriction. Furthermore, it is worth noting that researchers in the field sometimes assess not only the rank component of teachers’ judgment accuracy (i.e., the extent to which teachers’ judgments correlate with estimates of student achievement according to another criterion) but also a level and differentiation component (i.e., the extent to which teachers over- and underestimate students’ abilities and the extent to which teachers accurately assess the variance of students’ abilities, respectively) (see [61, 62]).

Erroneous estimates of the rank component stemming from artifacts imply that estimates of the level and differentiation components of teachers’ judgment accuracy will also be biased. In this study, correcting for measurement errors meaningfully increased estimates of teachers’ judgment accuracy (from *r*_*ob*_ = .58 up to ρ = .65). Our study demonstrates the importance of employing psychometric rather than classical meta-analytic approaches to synthesize research findings on a specific subject. Furthermore, our findings illustrate that neglecting to address artifacts could result in an underestimation of replication success.

Furthermore, to the best of our knowledge, our meta-analysis of teachers’ judgment accuracy appears to be the first wherein teachers’ judgment is regarded as a measurement instrument. This perspective emphasizes the necessity of applying psychometric corrections to teachers’ judgments, akin to those applied to other measurement instruments. This approach is not entirely new; in their book *Noise*, Kahneman and colleagues [63] introduced a chapter titled “Your Mind is a Measurement Instrument,” in which they noted that “Judgment can therefore be described as measurement in which the instrument is a human mind” (p. 39). Thus, we contend that teachers’ judgments can also be conceptualized and evaluated as a measurement instrument.

Our results also contribute new insights into the moderators influencing teachers’ judgment accuracy. Consistent with Südkamp et al. [22], we found no evidence that the accuracy of teachers’ judgments varied based on subject area. In our analyses, we also found no evidence that teachers’ judgment accuracy depended on student gender, either across different subjects or within specific subjects. However, it is important to note that our inability to investigate the potential moderating effect of teachers’ gender was due to missing data. Therefore, we propose that future studies and reviews should prioritize examining both teacher and student gender as potential moderator(s) of teachers’ judgment accuracy. In addition, we point out that future meta-analyses should also consider our moderator analyses on gender and their group categorization, which was useful for our dataset, but needs to be verified in future analyses with a larger dataset that includes teachers’ gender, as our results still show large heterogeneity which could not be explained by our presented moderator analyses.

Despite correcting for artifacts increasing estimates of teachers’ judgment accuracy, our results nevertheless suggest that there is still considerable room to improve teachers’ judgment accuracy (see, e.g., [64, 65]). In the present study, we found that estimates of judgment accuracy were highly similar in the available samples of classical and lens model judgment studies. In our previous analysis of lens model studies [24, 28], we highlighted the significance of the knowledge component within the lens model equation as a key determinant of teachers’ judgment accuracy. Hence, enhancing this knowledge component—potentially through teacher training initiatives aimed at augmenting teachers’ understanding of how to interpret various cues pertaining to students’ academic achievement—may enhance teachers’ judgment accuracy.

### Limitations and suggestions for future research

As previously mentioned, the absence of data presented a significant challenge in conducting the current study. Regrettably, insufficient data were available to facilitate an individual person data (IPD) meta-analysis of classical teachers’ judgment accuracy studies. Consequently, we were unable to assess whether aggregation bias skewed the results of previous reviews. Furthermore, although we conducted the analysis according to current best practices, meta-analytic techniques continue to develop. Therefore, we encourage researchers to compile IPD, reliability values, and lens model data, and to regularly conduct updated reviews. Although our current study did not uncover evidence of publication bias, it remains a potential factor influencing the outcomes of meta-analyses in other fields. Consistent with Schmidt and Hunter’s [8] recommendation, we advocate that researchers conduct publication bias estimates, akin to those undertaking in our study.

It is worth noting that our review specifically targeted *teachers*’ judgment accuracy. Parents and other educational experts may sometimes judge students’ progress and potential more accurately than teachers (e.g., in reading [66]). Drawing upon the concept of the *wisdom of the crowd* phenomenon [67, 68], incorporating input from parents or other educational experts could enhance teachers’ abilities to assess their students’ academic abilities more accurately. Subsequent research should address whether aggregated judgments of students’ academic achievement derived from multiple sources, such as teachers, school counselors, or parents, yield more accurate judgments compared to judgments from a single source, such as a teacher.

## Supporting information

S1 File Literature search. (DOCX)

S2 File Studies included in our meta-analysis. (DOCX)

S3 File Coding. (DOCX)

S4 File Study characteristics. (DOCX)

S5 File Reliability. (DOCX)

S6 File Outlier diagnostics. (DOCX)
