# Supplementary material for: Teachers’ judgment accuracy: A replication check by psychometric meta-analysis
Source: PLoS One. 2024 Jul 25;19(7):e0307594. doi: 10.1371/journal.pone.0307594 (PMC11271880; doi:10.1371/journal.pone.0307594)
Supplement: S1 File — (DOCX) [file pone.0307594.s001.docx]

**Supplement 1: S1**

### **Literature search**

Südkamp et al. (2012) identified all studies published up until 2009; hence, our literature search covered 2009 to the start of 2018. We began by using Google scholar to search for all articles that had cited Südkamp et al. (2012) or Hoge and Coladarci (1989). As of 2017, 226 articles had cited Südkamp et al. (2012), and 378 articles had cited Hoge and Coladarci (1989). Using the same search terms as Südkamp et al. (2012), we then conducted a detailed keyword search of PsycInfo, ERIC, and the Web of Science (see S1 Table). We identified 188 relevant articles in PsycInfo, 430 in ERIC, and 314 in the Web of Science. In sum, considering all search strategies, we checked the titles and abstracts of 1,536 articles for eligibility. We used the same inclusion criteria as Südkamp et al. (2012, p. 747). Specifically, we included only studies that focused on students’ academic achievement and had an achievement test as an evaluation criterion; all studies measuring other abilities or characteristics (e.g., motivation) or using other criterion (e.g., grades) were excluded. We focused only on students in kindergarten through grade 12 and excluded all studies considering prekindergarten children, college students, or vocational training students.

We cross-checked our database with the database of studies included in the meta-analysis by Machts et al. (2016) to ensure that we only included studies focused specifically on students’ academic as opposed to cognitive competencies^^[[1]](#footnote-2)^^. The final database of “new” studies (NEW) consisted of 26 articles marked with “&” in the reference list. Please note that Johansson (2012) and Johansson (2015) were based on the same data; hence, only Johansson (2012) was included in NEW.

**S1 Table. Key words used in our database research.**

**S1 Fig. PRISMA flow chart of the literature search.**


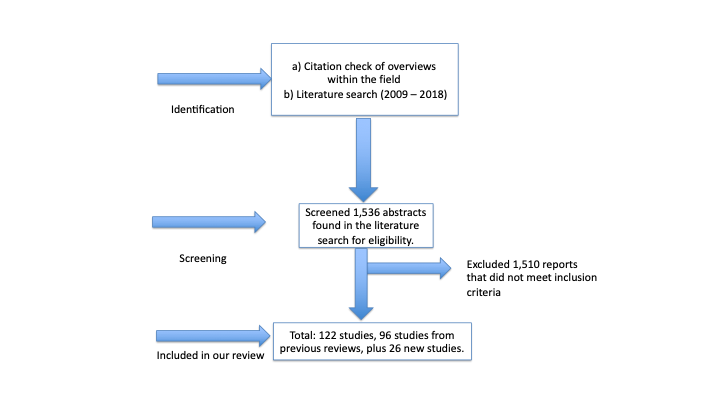


1. We underscore that two studies are included in Machts et al. (2016) as well as in our database because the study-authors considered students’ academic achievement (see Eaves, Campbell-Whatley, Dunn, Reilly, & Tate-Braxton, 1994; Miller, & Davis, 1992). [↑](#footnote-ref-2)
