# Supplementary material for: Teachers’ judgment accuracy: A replication check by psychometric meta-analysis
Source: PLoS One. 2024 Jul 25;19(7):e0307594. doi: 10.1371/journal.pone.0307594 (PMC11271880; doi:10.1371/journal.pone.0307594)
Supplement: S2 File — (DOCX) [file pone.0307594.s002.docx]

**Supplement 2: S2**

### **Studies included in our meta-analysis**

* Studies included in the meta-analysis by Hoge and Coladarci (1989)

% Studies included in the meta-analysis by Südkamp et al. (2012)

# Studies included in the meta-analysis by Kaufmann et al. (2013)

& Studies included in our new database

Totally, the dataset considered 122 studies.

1. %Anders, Y, Kunter, M., Brunner, M, Krauss, S., & Baumert, J. (2010). Diagnostische Fähigkeiten von Mathematiklehrkräften und ihre Wirkungen auf auf die Leistungen ihrer Schülerinnen und Schüler (Mathematics teachers’ diagnostic skills and their impact on students’ achievement). *Psychologie in Erziehung und Unterricht, 57*, 175–193. <http://dx.doi.org/10.2378/peu2010.art13d>
2. *Airasian, P. W., Kellaghan, T., Madaus, G. F., & Pedulla, J. J. (1977). Proportion and direction of teacher rating changes of pupils' progress attributable to standardized test information. *Journal of Educational Psychology, 69*, 702–709.
3. #Athanasou, J. A., & Cooksey, R. W. (2001). Judgment of factors influencing interest: An Australian study. *Journal of Vocational Education Research, 26*(1), 77–96.
4. %Bailey, A. L., & Drummond, K. V. (2006). Who is at risk and why? Teachers’ reasons for concern and their understanding and assessment of early literacy. *Educational Assessment, 11*, 149–178. <https://doi.org/10.1080/10627197.2006.9652988>
5. &Baker, C. N., Tichovolsky, M. H., Kupersmidt, J. B., Voegler-Lee, M. E., & Arnold, D. H. (2015). Teacher (mis)perceptions of preschoolers’ acadmic skills: Predictors and associations with longitudinal outcomes. *Journal of Educational Psychology, 107*(3), 805–820. https://doi.org/[10.1037/edu0000008](https://dx.doi.org/10.1037%2Fedu0000008)
6. %Bang, H. J., Suarez-Orozco, C., Pakes, J., & O’Conner, E. (2009). The importance of homework in determining immigrants students’ grades in schools in the USA context. *Educational Research, 51,* 1–25. <https://doi.org/10.1080/00131880802704624>
7. %Bates, C., & Nettelbeck, T. (2001). Primary school teachers’ judgements of reading achievement*. Educational Psychology, 21*, 177–187. <https://doi.org/10.1080/01443410020043878>
8. %Begeny, J. C., Eckert, T. L., Montarello, S. A., & Storie, M. S. (2008). Teachers’ perceptions of students’ reading abilities: An examination of the relationship between teachers’ judgments and students’ performance across a continuum of rating methods. *School Psychology Quarterly*, *23*, 43–55. <https://doi.org/10.1037/1045-3830.23.1.43>
9. &Begeny, J. C., Krouse, H. E., Brown, K. G., & Mann, C. M. (2011). Teacher judgment of students’ reading abilities across a continuum of rating methods and achievement measures. *School Psychology Review, 40*(1), 23–28.
10. &Behrmann, L., & Souvignier, E. (2013). The relation between teachers’ diagnostic sensitivity, their instructional activities, and their students’ achievement gains in reading. *Zeitschrift für Pädagogische Psychologie, 27*(4), 283–293.
11. %Benner, A. D., & Mistry, R. S. (2007). Congruence of mother and teacher educational expectations and low-income youth’s academic competence. *Journal of Educational Psychology*, *99*, 140–153. <https://doi.org/10.1037/0022-0663.99.1.140>
12. %Beswick, J. F., Willms, J. D., & Sloat, E. A. (2005). A comparative study of teacher ratings of emergent literacy skills and student performance on a standardized measure. *Education, 126,* 116–137.
13. %Burns, M. K., & Symington, T. (2003). A comparison of the spontaneous writing quotient of the Test of Written Language (3rd ed.) and teacher ratings of writing progress. *Assessment for Effective Intervention, 28*, 29–34. <https://doi.org/10.1177/073724770302800203>
14. &Bressoux, P., & Pansu, P. (2016). Pupils’ self-perceptions: The role of teachers’ judgment controlling for big-fish-little-pond effect. *European Journal of Psychology of Education, 31*, 341–357. <https://doi.org/10.1007/s10212-015-0264-7>
15. &Carmichael, C. (2015). Discrepancies between standardized testing and teacher judgments in an Australian primary school context. *Mathematics Teacher Education and Development, 17*(1), 62–75.
16. *Coladarci, T. (1986). Accuracy of teacher judgments of student responses to standardized test items. *Journal of Educational Psychology, 78*(2), 141–146.<https://doi.org/10.1037/0022-0663.78.2.141>
17. #Cooksey, R. W., Freebody, P., & Davidson, G. R. (1986). Teachers’ predictions of children’s early reading achievement: An application of social judgment theory. *American Educational Research* *Journal, 23*(1)*,* 41–64. <https://doi.org/10.3102/00028312023001041>
18. #Cooksey, R.W., Freebody, P., & Wyatt-Smith, C. (2007). Assessment as judgment-in-context: Analyzing how teachers evaluate students' writing. *Educational Research and Evaluation, 13*(5), 401–434. <https://doi.org/10.1080/13803610701728311>
19. %Dale, P. S., Harlaar, N., & Plomin, R. (2005). Telephone testing and teacher assessment of reading skills in 7-year-olds: I. Substantial correspondence for a sample of 5,544 children and for extremes. *Reading and Writing, 18*, 385– 400. <https://doi.org/10.1007/s11145-004-8130-z>
20. %Demaray, M. K., & Elliott, S. N. (1998). Teachers’ judgments of students’ academic functioning: A comparison of actual and predicted performances. *School Psychology Quarterly, 13*, 8–24. <https://doi.org/10.1037/h0088969>
21. %DiPerna, J. C., & Elliott, S. N. (1999). Development and validation of the Academic Competence Evaluation Scales. *Journal of Psychoeducational Assessment, 17*, 207–225. <https://doi.org/10.1177/073428299901700302>
22. *Doherty, J., & Conolly, M. (1985). How accurately can primary school teachers predict the scores of their pupiles in standardised tests of attainment? A study of some non-cognitive factors that influence specific judgments. *Educational Studies, 11*, 41–60. <https://doi.org/10.1080/0305569850110105>
23. %Dompnier, B., Pansu, P., & Bressoux, P. (2006). An integrative model of scholastic judgments: Pupils’ characteristics, class context, halo effect and internal attributions. *European Journal of Psychology of Education, 21*, 119–133. <https://doi.org/10.1007/BF03173572>
24. %DuPaul, G. J., Rapport, M. D., & Perriello, L. M. (1991). Teacher ratings of academic skills: The development of the Academic Performance Rating Scale. *School Psychology Review, 20,* 284–300.
25. %Eaves, R. C., Campbell-Whatley, G., Dunn, C., Reilly, A. S., & Tate- Braxton, C. (1994). Comparison of the Slosson Full-Range Intelligence Test and teacher judgments as predictors of students’ academic achievement. *Journal of Psychoeducational Assessment, 12*, 381–392. <https://doi.org/10.1177/073428299401200408>
26. %Eaves, R. C., Williams, P., Winchester, K., & Darch, C. (1994). Using teacher judgment and IQ to estimate reading and mathematics achievement in a remedial-reading program. *Psychology in the Schools, 31*, 261–272. [https://doi.org/10.1002/1520-6807(199410)31:4<261::AID-PITS2310310403>3.0.CO;2-K](https://doi.org/10.1002/1520-6807(199410)31:4%3c261::AID-PITS2310310403%3e3.0.CO;2-K)
27. %Eckert, T. L., Dunn, E. K., Codding, R. S., Begeny, J. C., & Kleinmann, A. E. (2006). Assessment of mathematics and reading performance: An examination of the correspondence between direct assessment of student performance and teacher report. *Psychology in the Schools, 43*, 247–265. <https://doi.org/10.1002/pits.20147>
28. %Elliott, J., Lee, S. W., & Tollefson, N. (2001). A reliability and validity study of the dynamic indicators of basic early literacy skills– modified. *School Psychology Review, 30*, 33–49. <https://doi.org/10.1080/02796015.2001.12086099>
29. %Eshel, Y., & Benski, M. (1995). Group-administered school readiness test and kindergarten teacher ratings as predictors of academic success in the first grade. *Megamot, 36,* 451–464.
30. #^*^Eshleman, J. L. (Unpublished). *Math teachers’ intuitions about student success in algebra.* (Unpublished doctoral dissertation in 2009). Oklahoma State University, Oklahoma, US.
31. %Espin, C., Shin, J., Deno, S. L., Skare, S., Robinson, S., & Benner, B. (2000). Identifying indicators of written expression proficiency for mid- dle school students. *The Journal of Special Education, 34,* 140–153. <https://doi.org/10.1177/002246690003400303>
32. *Farr, R., & Roelke, P. (1971). Measuring subskills of reading: Intercorrelations between standardized reading tests, teachers' ratings, and reading specialists' ratings. *Journal of Educational Measurement, 8*, 27–32. <https://doi.org/10.1111/j.1745-3984.1971.tb00903.x>
33. %Feinberg, A. B., & Shapiro, E. S. (2003). Accuracy of teacher judgments in predicting oral reading fluency. *School Psychology Quarterly, 18*, 52–65. <https://doi.org/10.1521/scpq.18.1.52.20876>
34. %Feinberg, A. B., & Shapiro, E. S. (2009). Teacher accuracy: An examination of teacher-based judgments of students’ reading with differing achievement level*. Journal of Educational Research, 102*, 453–462. <https://doi.org/10.3200/JOER.102.6.453-462>
35. &Fleckenstein, J., Leucht, M., & Köller, O. (2018). Teachers’ judgment accuracy concerning CEFR levels of prospective university students. *Language Assessment Quarterly, 15*(1), 90–101. <https://doi.org/10.1080/15434303.2017.1421956>
36. *%*Fletcher, J., Tannock, R., & Bishop, D. V. M. (2001). Utility of brief teacher rating scales to identify children with educational problems: Experience with an Australian sample. *Australian Journal of Psychology, 53*, 63–71. <https://doi.org/10.1080/00049530108255125>
37. %Flynn, J. M., & Rahbar, M. H. (1998). Improving teacher prediction of children at risk for reading failure. *Psychology in the Schools, 35*, 163–172. [https://doi.org/10.1002/(SICI)1520-6807(199804)35:2<163::AID-PITS8>3.0.CO;2-Q](https://doi.org/10.1002/(SICI)1520-6807(199804)35:2%3c163::AID-PITS8%3e3.0.CO;2-Q)
38. &Furnari, E. C., Whittaker, J., Kinzie, M., & DeCoster, J. (2017). Factors associated with accuracy in prekindergarten teacher ratings of students’ mathematics skills. *Journal of Psychoeducational Assessment, 35*(4), 410–423. <https://doi.org/10.1177/0734282916639195>
39. %Freeman, J. G. (1993). Two factors contributing to elementary school teachers’ predictions of students’ scores on the Gates–MacGinitie Reading Test, Level D. *Perceptual and Motor Skills, 76*, 536–538. <https://doi.org/10.2466/pms.1993.76.2.536>
40. %Gallant, D. J. (2009). Predictive validity evidence for an assessment program based on the Work Sampling System in mathematics and language and literacy. *Early Childhood Research Quarterly, 24,* 133–141. <https://doi.org/10.1016/j.ecresq.2009.03.003>
41. %Gottesman, R. L., Cerullo, F. M., Bennett, R. E., & Rock, D. A. (1991). Predictive validity of a screening test for mild school learning difficulties. *Journal of School Psychology, 29*, 191–205. <https://doi.org/10.1016/0022-4405(91)90001-8>
42. %Graney, S. B. (2008). General education teacher judgments of their low-performing students’ short-term reading progress. *Psychology in the Schools, 45*, 537–549. <https://doi.org/10.1002/pits.20322>
43. %Graue, M. E., & Shepard, L. A. (1989). Predictive validity of the Gesell School Readiness Tests. *Early Childhood Research Quarterly, 4,* 303–315. <https://doi.org/10.1016/0885-2006(89)90016-1>
44. %Gresham, F. M., MacMillan, D. L., & Bocian, K. M. (1997). Teachers as “tests”: Differential validity of teacher judgments in identifying students at-risk for learning difficulties. *School Psychology Review, 26*, 47–60. <https://doi.org/10.1080/02796015.1997.12085847>
45. *Gresham, F. M., Reschly, D. J., & Carey, M. P. (1987). Teachers as "tests": Classification accuracy and concurrent validation in the identification of learning disabled children. *School Psychology Review, 16*, 543–533. <https://doi.org/10.1080/02796015.1987.12085315>
46. %Gullo, D. F. (1990). Kindergarten schedules: Effects on teachers’ ability to assess academic achievement. *Early Childhood Research Quarterly, 5*, 43–51. <https://doi.org/10.1016/0885-2006(90)90005-L>
47. &Hammes, P. S., Bigras, M., & Aparecida Crepaldi, M. (2016). Validity and bias of academic achievement measures in the first year of elementary school. *International Journal of Research & Method in Education, 39*(1), 3–18, https://doi.org/10.1080/1743727X.2014.933473
48. %Hartman, J. M., & Fuller, M. L. (1997). The development of curriculum- based measurement norms in literature-based classrooms. *Journal of School Psychology, 35,* 377–389. <https://doi.org/10.1016/S0022-4405(97)00013-7>
49. %Hauser-Cram, P., Sirin, S. R., & Stipek, D. (2003). When teachers’ and parents’ values differ: Teachers’ ratings of academic competence in children from low-income families. *Journal of Educational Psychology, 95,* 813–820. <https://doi.org/10.1037/0022-0663.95.4.813>
50. %Hecht, S. A., & Greenfield, D. B. (2002). Explaining the predictive accuracy of teacher judgments of their students’ reading achievement: The role of gender, classroom behavior, and emergent literacy skills in a longitudinal sample of children exposed to poverty. *Reading and Writing, 15*, 789–809. <https://doi.org/10.1023/A:1020985701556>
51. *Helmke, A., & Schrader, F.-W. (1987). Interactional effects of instructional quality and teacher judgment accuracy on achievement. *Teaching and Teacher Education, 3*, 91–98. <https://doi.org/10.1016/0742-051X(87)90010-2>
52. %Helwig, R., Anderson, L., & Tindal, G. (2001). Influence of elementary student gender on teachers’ perceptions of mathematics achievement. *Journal of Educational Research, 95*, 93–102. https://doi.org/10.1080/ 00220670109596577
53. %Herbert, J., & Stipek, D. (2005). The emergence of gender differences in children’s perceptions of their academic competence. *Journal of Applied Developmental Psychology, 26*, 276–295. https://doi.org/10.1016/j.appdev.2005.02.007
54. %Hinnant, J. B., O’Brien, M., & Ghazarian, S. R. (2009). The longitudinal relations of teacher expectations to achievement in the early school year. *Journal of Educational Psychology, 101*, 662–670. <https://doi.org/10.1037/a0014306>
55. *Hoge, R. D., & Butcher, R. (1984). Analysis of teacher judgments of pupil achievement levels. *Journal of Educational Psychology, 76*(5), 777–781. [https://doi.org/10.1037/0022-0663.76.5.777](file:///Users/esther_kaufmann/Desktop/Esther%20Kaufmann%20-%20Artikel_Meta-Analyse_Urteilsgenauigkeit/Article/SubmissionPB/%20https:/doi.org/10.1037/0022-0663.76.5.777)
56. %Hodges, C. A. (1997). How valid and useful are alternative assessments for decision-making in primary grade classrooms? *Reading Research and Instruction, 36,* 157–173. <https://doi.org/10.1080/19388079709558235>
57. *Hopkins, K. D., Dobson, J. C., & Oldridge, O. A. (1962). The concurrent and congruent validities of the Wide Range Achievement Test. *Educational and Psychological Measurement, 22*, 791–793. <https://doi.org/10.1111/j.1745-3984.1985.tb01056.x>
58. *Hopkins, K. D., George, C. A., & Williams, D. D. (1985). The concurrent validity of standardized achievement tests by content area using teachers' rating as criteria. *Journal of Educational Measurement, 22*, 177–182. <https://doi.org/10.1111/j.1745-3984.1985.tb01056.x>
59. %Hughes, J. N., Gleason, K. A., & Zhang, D. A. (2005). Relationship influences on teachers’ perceptions of academic competence in academically at-risk minority and majority first grade students. *Journal of School Psychology, 43,* 303–320. <https://doi.org/10.1016/j.jsp.2005.07.001>
60. %Jenkins, J. R., & Jewell, M. (1993). Examining the validity of two measures for formative teaching: Reading aloud and maze. *Exceptional Children, 59*, 421–432. [https://doi.org/10.1177/001440299305900505](https://doi.org/10.1177%2F001440299305900505)
61. &Johansson, S., Myrberg, E., & Rosén, M. (2012) Teachers and tests: assessing pupils' reading achievement in primary schools. *Educational Research and Evaluation, 18*(8), 693–711, https://doi.org/10.1080/13803611.2012.718491
62. %Jorgenson, C. B., Jorgenson, D. E., Gillis, M. K., & McCall, C. M. (1993). Validation of a screening instrument for young children with teacher assessment of school performance. *School Psychology Quarterly, 8*, 125–139. <https://doi.org/10.1037/h0088834>
63. &Kaiser, J., Retelsdorf, J., Südkamp, A., & Möller, J. (2013). Achievement and engagement: How student characteristics influence teacher judgments. *Learning and Instruction, 28*, 73–84. <https://doi.org/10.1016/j.learninstruc.2013.06.001>
64. &Kaiser, J., Südkamp, A., & Möller, J. (2017). The effects of student characteristics on teachers’ judgment accuracy: Disentangling ethnicity, minority status, and achievement. *Journal of Educational Psychology, 109*(6), 871–888. https://doi.org/[10.1037/edu0000156](https://doi.org/10.1037/edu0000156)
65. %Karing, C. (2009). Diagnostische Kompetenz von Grundschul- und Gymnasiallehrkräften im Leistungsbereich und im Bereich Interessen [Diagnostic competence of elementary and secondary school teachers in the domains of competence and interests]. *Zeitschrift für Pädagogische Psychologie/German Journal of Educational Psychology, 23*, 197–209. <https://doi.org/10.1024/1010-0652.23.34.197>
66. *%Karing, C., Matthäi, J., & Artelt, C. (2011). Lower secondary school teacher judgment accuracy of students’ reading competence – A matter of specificity? *Zeitschrift für Pädagogische Psychologie/German Journal of Educational Psychology, 25*(3), 159–172. <https://doi.org/10.1024/1010-0652/a000041>
67. %Kenealy, P., Frude, N., & Shaw, W. (1991). Teacher expectations as predictors of academic success*. Journal of Social Psychology, 131*, 305–306. <https://doi.org/10.1080/00224545.1991.9713856>
68. %Kenny, D. T., & Chekaluk, E. (1993). Early reading performance: A comparison of teacher-based and test-based assessments. *Journal of Learning Disabilities, 26*, 227–236. https://doi.org/10.1177/002221949302600403
69. *Kikas, E., Silinskas, G., & Soodla, P. (2015). The effects of children’s reading skills and interest on teacher perceptions of children’s skills and individualized support. *International Journal of Behavioral Development, 39*(5), 402–412. [https://doi.org/10.1177/0165025415573641](https://doi.org/10.1177%2F0165025415573641)
70. &Kikas, E., Soodla, P., & Mägi, K. (2018). Teacher judgments of student reading and math skills: Associations with child- and classroom-related factors. *Scandinavian Journal of Educational Research, 62*(5), 783–797. https://doi.org/10.1080/00313831.2017.1307271
71. %Kuklinski, M. R., & Weinstein, R. S. (2001). Classroom and develop- mental differences in a path model of teacher expectancy effects. *Child Development, 72*, 1554–1578. <https://doi.org/10.1111/1467-8624.00365>
72. %Kwok, D. C., & Lytton, H. (1996). Perceptions of mathematics ability versus actual mathematics performance: Canadian and Hong Kong Chinese children. *British Journal of Educational Psychology, 66,* 209–222. <https://doi.org/10.1111/j.2044-8279.1996.tb01190.x>
73. *Leinhardt, G. (1983). Novice and expert knowledge of individual student's achievement. *Educational Psychologist, 18*, 165–179. <https://doi.org/10.1080/00461528309529272>
74. %Lembke, E. S., Foegen, A., Whittaker, T. A., & Hampton, D. (2008). Establishing technically adequate measures of progress in early numeracy. *Assessment for Effective Intervention, 33*, 206–214. <https://doi.org/10.1177/1534508407313479>
75. &Leucht, M., Tiffin-Richards, S., Vock, M., Pant, H. A., & Köller, O. (2012). English teachers’ diagnostic skills in judging their students’ competencies on the basis of the Common European Framework if Reference. *Zeitschrift für Entwicklungspsychologie und Pädagogische Psychologie, 44*(4), 163–177. https://doi.org/10.1026/0049-8637/a000071
76. %Li, H., Pfeiffer, S. I., Petscher, Y., Kumtepe, A. T., & Mo, G. (2008). Validation of the gifted rating scales–school form in China. *Gifted Child Quarterly, 52,* 160–169. <https://doi.org/10.1177/0016986208315802>
77. %Limbos, M. M., & Geva, E. (2001). Accuracy of teacher assessments of second-language students at risk for reading disability. *Journal of Learning Disabilities, 34*, 136–151. <https://doi.org/10.1177/002221940103400204>
78. %Lorenz, C., & Artelt, C. (2009). Fachspezifität und Stabilität diagnostischer Kompetenz von Grundschullehrkräften in den Fächern Deutsch und Mathematik [Domain specificity and stability of diagnostic competence among primary school teachers in the school subjects of German and Mathematics]. *Zeitschrift für Pädagogische Psychologie, 23*(3-4), 211–222. <https://doi.org/10.1024/1010-0652.23.34.211>
79. *Luce, S. R., & Hoge, R. D. (1978). Relations among teacher rankings, pupil-teacher interactions, and academic achievement: A test of the teacher expectancy hypothesis. *American Educational Research Journal, 15*, 489–500. <https://doi.org/10.3102/00028312015004489>
80. %Madelaine, A., & Wheldall, K. (2005). Identifying low-progress readers: Comparing teacher judgment with a curriculum-based measurement procedure. *International Journal of Disability, Development, and Education, 52*, 33–42. <https://doi.org/10.1080/10349120500071886>
81. %Madon, S., Smith, A., Jussim, L., Russell, D. W., Eccles, J., Palumbo, P., & Walkiewicz, M. (2001). Am I as you see me or do you see me as I am? Self-fulfilling prophecies and self-verification. *Personality and Social Psychology Bulletin, 27*, 1214–1224. <https://doi.org/10.1177/0146167201279013>
82. %Maguin, E., & Loeber, R. (1996). How well do ratings of academic performance by mothers and their sons correspond to grades, achievement test scores, and teachers’ ratings? *Journal of Behavioral Education, 6,* 405–425. <https://doi.org/10.1007/BF0211051>
83. %Martinez, J. F., Stecher, B., & Borko, H. (2009). Classroom assessment practices, teacher judgments, and student achievement in mathematics: Evidence from the ECLS. *Educational Assessment, 14,* 78–102. <https://doi.org/10.1080/10627190903039429>
84. %Maunganidze, L., Ruhode, N., Shoniwa, L., Kasayira, J. M., Sodi, T., & Nyanhongo, S. (2008). Teacher ratings and standardized test scores: How good for predicting achievement in students with learning support placement? *Journal of Psychology in Africa, 18*, 255–258. <https://doi.org/10.1080/14330237.2008.10820194>
85. %McElvany, N., Schroeder, S., Hachfeld, A., Baumert, J., Richter, T., Schnotz, w., & Ullrich, M. (2009). Diagnostische Fähigkeiten von Lehrkräften bei der Einschätzung von Schülerleistungen und Aufgabenschwierigkeiten bei Lernmedien mit instruktionalen Bildern [Teachers’ diagnostic skills to judge student performance and task difficulty when learning materials include instructional pictures]. *Zeitschrift für Pädagogische Psychologie/German Journal of Educational Psychology, 23*, 223–235. <https://doi.org/10.1024/1010-0652.23.34.223>
86. %Meisels, S. J., Bickel, D. D., Nicholson, J., Xue, Y., & Atkins-Burnett, S. (2001). Trusting teachers’ judgments: A validity study of a curriculum- embedded performance assessment in kindergarten to grade 3. *American Educational Research Journal, 38*, 73–95. <https://doi.org/10.3102/00028312038001073>
87. &Meissel, K., Meyer, F., Yao, E. S., & Rubie-Davies, C. M. (2017). Subjectivity of teacher judgments: Exploring student characteristics that influence teacher judgments of student ability. *Teaching and Teacher Education, 65*, 48–60. <https://doi.org/10.1016/j.tate.2017.02.021>
88. %Methe, S. A., Hintze, J. M., & Floyd, R. G. (2008). Validation and decision accuracy of early numeracy skill indicators. *School Psychology Review, 37*, 359–373.
89. %Meyer, M., Wilgosh, L., & Mueller, H. (1990). Effectiveness of teacher- administered tests and rating scales in predicting subsequent academic performance. *Alberta Journal of Educational Research, 36*, 257–264.
90. %Miller, S. A., & Davis, T. L. (1992). Beliefs about children: A comparative study of mothers, teachers, peers, and self. *Child Development, 63,* 1251–1265. <https://doi.org/10.2307/1131531>
91. %Montague, M., Enders, C., & Castro, M. (2005). Academic and behavioral outcomes for students at risk for emotional and behavioral disorders. *Behavioral Disorders, 31,* 84–94. [https://doi.org/10.1177/019874290503100106](https://doi.org/10.1177%2F019874290503100106)
92. *Oliver, J. E., & Arnold, R. D. (1978). Comparing a standardized test, an informal inventory and teacher judgment on third grade reading. *Reading Improvement, 15*, 56–59.
93. &Paleczek, L., Seifert, S., & Gasteiger-Klicpera, B. (2017). Influences on teachers’ judgment accuracy of reading abilities on second and third grade students: A multilevel analysis. *Psychology in the Schools, 54*(3), 228–245. <https://doi.org/10.1002/pits.21993>
94. *Pedulla, J. J., Airasian, P. W., & Madaus, G. F. (1980). Do teacher ratings and standardized test results of students yield the same information? *American Educational Research Journal, 17*, 303–307. <https://doi.org/10.3102/00028312017003303>
95. %Pomplun, M. (2004). The differential predictive validity of the initial skills analysis: Reading screening tests for K-3. *Educational and Psychological Measurement, 64*, 813–827. <https://doi.org/10.1177/0013164404263879>
96. &Rausch, T., Karing, C., Dörfler, T., & Artelt, C. (2016). Personality similarity between teachers and their students influences teacher judgement of student achievement. *Educational Psychology, 36*(5), 863–878. <https://doi.org/10.1080/01443410.2014.998629>
97. &Rausch, T., Matthäi, J., & Artelt, C. (2015). Teacher knowledge and judgment accuracy in the domain of text comprehension. *Zeitschrift für Entwicklungspsychologie und Pädagogische Psychologie, 47*(3), 147–158. <https://doi.org/10.1026/0049-8637/a000124>
98. %Saint-Laurent, L., Hébert, M., Royer, É., & Piérard, B. (1997). Identification of students with academic difficulties: Implications for research and practice. *Canadian Journal of School Psychology, 12*, 143–154. <https://doi.org/10.1177/082957359701200211>
99. %Salvesen, K. A., & Undheim, J. O. (1994). Screening for learning dis- abilities with teacher rating scales. *Journal of Learning Disabilities, 27*, 60–66. <https://doi.org/10.1177/002221949402700109>
100. %Schrader, F.-W., & Helmke, A. (1990). Lassen sich *Lehrer bei der Leistungsbeurteilung* von sachfremden Gesichtspunkten leiten? Eine Untersuchung zu Determinanten diagnostischer Lehrerurteile [Are teachers influenced by extrinsic factors when evaluating scholastic per- formance? A study on the determinants of teachers’ judgments]. *Zeitschrift für Entwicklungspsychologie und Pädagogische Psychologie, 22*, 312–324.
101. *Sharpley, C. F., & Edgar, E. (1986). Teachers' ratings vs standardized tests: An empirical investigation of agreement between two indices of achievement. *Psychology in the Schools, 23*, 106–111. [https://doi.org/10.1002/1520-6807(198601)23:1<106::AID-PITS2310230117>3.0.CO;2-C](https://doi.org/10.1002/1520-6807(198601)23:1%3c106::AID-PITS2310230117%3e3.0.CO;2-C)
102. *Silverstein, A. B., Brownlee, L., Legutki, G., & MacMillan, D. L. (1983). Convergent and discriminant validation of two methods of assessing three academic traits. *Journal of Special Education, 17*, 63–68. https://doi.org/[10.1177/002246698301700108](http://dx.doi.org/10.1177/002246698301700108)
103. %Sink, C. A., Barnett, J. E., & Pool, B. A. (1993). Perceptions of scholastic competence in relation to middle-school achievement. *Perceptual and Motor Skills, 76*, 471–478. <https://doi.org/10.2466/pms.1993.76.2.471>
104. %Sofie, C. A., & Riccio, C. A. (2002). A comparison of multiple methods for the identification of children with reading disabilities. *Journal of Learning Disabilities, 35*, 234–244. <https://doi.org/10.1177/002221940203500305>
105. &Sorhagen, N. S. (2013). Early teacher expectations disproportionately affect poor children’s high school performance. *Journal of Educational Psychology, 105*(2), 465–477. [https://doi.org/10.1037/a0031754](https://psycnet.apa.org/doi/10.1037/a0031754)
106. &Stang, J., & Urhahne, D. (2016). Stabilität, Bezugsnormorientierung und Auswirkungen der Urteilsgenauigkeit [Stability, reference norm orientation, and effects of judgment accuracy]. *Zeitschrift für Pädagogische Psychologie / German Journal of Educational Psychology, 30*(4), 251–262. [https://doi.org/10.1024/1010-0652/a000190](https://psycnet.apa.org/doi/10.1024/1010-0652/a000190)
107. %Teisl, J. T., Mazzocco, M. M. M., & Myers, G. F. (2001). The utility of kindergarten teacher ratings for predicting low academic achievement in first grade. *Journal of Learning Disabilities, 34*, 286–293. <https://doi.org/10.1177/002221940103400308>
108. &Timmermans, A. C., Kuyper, H., van der Werf, G. (2015). Accurate, inaccurate, or biased teacher expectations: Do Dutch teachers differ in their expectations at the end of primary education? *British Journal of Educational Psychology, 85*, 459–478. [https://doi.org/ 10.1111/bjep.12087](https://doi.org/%2010.1111/bjep.12087)
109. %Tindal, G., & Marston, D. (1996). Technical adequacy of alternative reading measures as performance assessments. *Exceptionality, 6,* 201–230. <https://doi.org/10.1207/s15327035ex0604_1>
110. %Trautwein, U., & Baeriswyl, F. (2007). Wenn leistungsstarke Klassenkameraden ein Nachteil sind: Referenzgruppeneffekte bei Übertrittsentscheidungen [When high-achieving classmates put students at a disadvantage: Reference group effects at the transition to secondary schooling]. *Zeitschrift für Pädagogische Psychologie/German Journal of Educational Psychology, 21,* 119–133. <https://doi.org/10.1024/1010-0652.21.2.119>
111. %Triga, A. (2004). An analysis of teachers’ rating scales as sources of evidence for a standardised Greek reading test. *Journal of Research in Reading, 27*, 311–320. <https://doi.org/10.1111/j.1467-9817.2004.00234.x>
112. %van Kraayenoord, C. E., & Schneider, W. E. (1999). Reading achievement, metacognition, reading self-concept and interest: A study of German students in Grades 3 and 4. European *Journal of Psychology of Education, 14*, 305–324. <https://doi.org/10.1007/BF03173117>
113. &Valdez, A. (2013). Teacher judgment of reading achievement: Cross-sectional and longitudinal perspective. *Journal of Educational Learning, 2*(4), 186–200. <http://dx.doi.org/10.5539/jel.v2n4p186>
114. &Virinkoski, R., Lerkkanen, M.-K., Holopainen, L., Eklund, K., & Aro, M. (2018). Teachers’ ability to identify children at early risk for reading difficulties in Grade 1. *Early Childhood Education Journal, 46,* 497–509.
115. %Webster, R. E., Hewett, B., & Crumbacker, H. M. (1989). Criterion- related validity of the WRAT–R and K-TEA with teacher estimates of actual classroom academic performance. *Psychology in the Schools, 26*, 243–248. https://doi.org/10.1002/1520-6807(198907)26:3<243::AID- PITS2310260304>3.0.CO;2-M
116. #Wiggins, N., & Kohen, E. S. (1971). Man versus model of man revisited: The forecasting of graduate school success. *Journal of Personality and Social Psychology, 19*(1), 100–106. [https://doi.org/ 10.1037/h0031147](https://doi.org/%2010.1037/h0031147)
117. %Wilson, J., & Wright, C. R. (1993). The predictive validity of student self-evaluations, teachers’ assessments, and grades for performance on the Verbal Reasoning and Numerical Ability Scales of the Differential Aptitude Test for a sample of secondary school students attending rural Appalachia schools. *Educational and Psychological Measurement, 53*, 259–270. <https://doi.org/10.1177/0013164493053001029>
118. %Wilson, M. S., Schendel, J. M., & Ulman, J. E. (1992). Curriculum-based measures, teachers’ ratings, and group achievement scores: Alternative screening measures. *Journal of School Psychology, 30,* 59–76. <https://doi.org/10.1016/0022-4405(92)90020-6>
119. %Wright, C. R., & Houck, J. W. (1995). Gender differences among self- assessments, teacher ratings, grades, and aptitude test scores for a sample of students attending rural secondary schools. *Educational and Psychological Measurement, 55,* 743–752. <https://doi.org/10.1177/0013164495055005005>
120. *Wright, D., & Wiese, M. J. (1988). Teacher judgment in student evaluation: A comparison of grading methods. *Journal of Educational Research, 82*, 10–14. <https://doi.org/10.1080/00220671.1988.10885858>
121. &Zhou, J., & Urhahne, D. (2013). Teacher judgment, student motivation, and the mediating effect of attributions. *European Journal of Psychology of Education, 28*, 275–296. <https://doi.org/10.1007/s10212-012-0114-9>
122. &Zhu, M., & Urhahne, D. (2015). Teachers’ judgments of students’ foreign language achievement. *European Journal of Psychology of Education, 30*, 21–39. <https://doi.org/10.1007/s10212-014-0225-6>
