# Supplementary material for: Teachers’ judgment accuracy: A replication check by psychometric meta-analysis
Source: PLoS One. 2024 Jul 25;19(7):e0307594. doi: 10.1371/journal.pone.0307594 (PMC11271880; doi:10.1371/journal.pone.0307594)
Supplement: S3 File — (DOCX) [file pone.0307594.s003.docx]

**Supplement 3: S3**

**Coding**

We coded each study according to the following characteristics: publication year, origin (United States/Canada/ Australia/Europe/Other, including three studies from China and one study each from New Zealand, Israel, and Zimbabwe), whether individual-level data were available, teacher sample size (number of judges), student sample size (number of students judged), students’ grade level, the gender composition of the student sample, judgment subject area (Language/Math/Both language and math/Other), reliability of the judgments and the reliability type/measure (any /internal consistency reliability/Cronbach’s alpha), judgment criterion, reliability of the judgment criterion and reliability type/measure. Each study was coded by two people, and any coding differences were discussed until agreement was reached. The complete data set is available by request.
