# Supplementary material for: Teachers’ judgment accuracy: A replication check by psychometric meta-analysis
Source: PLoS One. 2024 Jul 25;19(7):e0307594. doi: 10.1371/journal.pone.0307594 (PMC11271880; doi:10.1371/journal.pone.0307594)
Supplement: S4 File — (DOCX) [file pone.0307594.s004.docx]

**Supplement 4: S4**

**Study characteristics**

**Publication year and origin**

S4 Fig displays the number of studies published by decade and origin. Only one study had been conducted in the 1960s (see Hopkins, Dobson, & Oldridge, 1962). As seen in S4 Fig, the number of published studies on teachers’ judgment accuracy has generally increased across time, although there seems to have been a slight decrease since 2009. Overall, most studies were from the United States (54%), followed by Europe (27%), Australia (7.3%), Canada (6.5%), and other regions (4.8%). Whereas earlier studies were mostly from the United States, more recent studies were mainly from Europe. Half of the studies conducted outside of North America, Australia, and Europe were published after 2010 (Meissel et al., 2017; Zho & Urhahne, 2015; Zhou & Urhahne, 2013).

**S4 Fig. Number of published studies on teachers’ judgment accuracy concerning students’ academic abilities by decade and origin (k = 122).**
