# Supplementary material for: Teachers’ judgment accuracy: A replication check by psychometric meta-analysis
Source: PLoS One. 2024 Jul 25;19(7):e0307594. doi: 10.1371/journal.pone.0307594 (PMC11271880; doi:10.1371/journal.pone.0307594)
Supplement: S5 File — (DOCX) [file pone.0307594.s005.docx]

**Supplement 5: S5**

**Reliability**

Specifically, information on the internal consistency reliability of teachers’ judgments was available for *k* = 39 studies (*k* = 29 studies reported Cronbach’s alpha and *k* = 10 studies reported unidentifiable internal consistency reliability values), while information on the internal consistency reliability of the criterion was available for *k* = 36 studies (*k* = 24 studies reported Cronbach’s alpha and *k* = 12 reported unidentifiable internal consistency reliability values), see S5 Table.

**S5 Table. Available reliability information.**

| Reliability information | Judgments | | | | |  | Criterion | | | | |
| --- | --- | --- | --- | --- | --- | --- | --- | --- | --- | --- | --- |
|  | *N* | *M* | *SD* | *min* | *max* |  | *N* | *m* | *SD* | *min* | *max* |
| Any type/measure | 47 | 0.89 | 0.09 | 0.67 | 0.99 |  | 71 | 0.86 | 0.08 | 0.64 | 0.99 |
| Internal consistency reliability^1^ | 39 | 0.91 | 0.07 | 0.67 | 0.99 |  | 37 | 0.84 | 0.08 | 0.66 | 0.99 |
| Cronbach’s alpha | 29 | 0.92 | 0.05 | 0.80 | 0.98 |  | 24 | 0.82 | 0.08 | 0.66 | 0.99 |

*Note.* ^1^Includes studies which reported Cronbach’s alpha as well as studies which reported an unidentifiable measure of internal consistency reliability. *N* = number of reliability values.
