# Supplementary material for: Teachers’ judgment accuracy: A replication check by psychometric meta-analysis
Source: PLoS One. 2024 Jul 25;19(7):e0307594. doi: 10.1371/journal.pone.0307594 (PMC11271880; doi:10.1371/journal.pone.0307594)
Supplement: S6 File — (DOCX) [file pone.0307594.s006.docx]

**Supplement 6: S6**

**Outlier diagnostics**

Based on the forest plot and influential case diagnostics, we identified three outlier studies: Graney (2008), Karing (2011), and Zhou (2013), see also S5 Fig. In all three studies, teachers’ judgment accuracy was *r* ≤ .2. We identified no outlier studies in either the subject or subject/gender subsamples.

**S6 Fig. Outlier diagnostic revealed three studies (marked by red dots) as outliers.**
